# Manuka honey in combination with azithromycin shows potential for improved activity against *Mycobacterium abscessus*

**DOI:** 10.1016/j.tcsw.2022.100090

**Published:** 2022-11-17

**Authors:** Victoria C. Nolan, James Harrison, Jonathan A.G. Cox

**Affiliations:** School of Life and Health Sciences, Aston University, Aston Triangle, Birmingham B4 7ET, UK

**Keywords:** Manuka honey, *Mycobacterium abscessus*, Synergy, Antimicrobial, Azithromycin

## Abstract

*Mycobacterium abscessus* is an increasingly prevalent opportunistic pathogen causing both pulmonary and skin and soft tissue infections. It is of increasing concern for immunocompromised individuals, such as those with cystic fibrosis, due to its highly drug resistant nature and ability to evade the host immune system. Current treatments for *M. abscessus* pulmonary infections are largely ineffective and treatment outcomes are generally poor, thus we urgently require new treatments to combat these infections. Recently, it has been demonstrated that manuka honey is effective against *M. abscessus* and can improve the inhibitory effect of amikacin. Here, we explore the potential improvement of both azithromycin and tobramycin with the addition of manuka honey against *M. abscessus* complex. Improved growth inhibition was observed for azithromycin with manuka honey against all *M. abscessus* subspecies. Improved bactericidal activity was also observed. Importantly, the macrolide resistant *M. abscessus* subsp. *bolletii* showed improved inhibition and bactericidal activity was obtained in response to 0.117 g/mL manuka honey MGO40 with 16 µg/mL azithromycin. No improved activity was observed for tobramycin and manuka honey against any of the *M. abscessus* isolates tested. This demonstrates the potential for antibiotic enhancement by the addition of manuka honey, furthering the applications of therapeutic manuka honey.

## 1 Introduction

*Mycobacterium abscessus* is a rapidly growing non-tuberculous mycobacteria of increasing concern, with the ability to cause pulmonary infections as well as infections of the skin and soft tissue (Abdelaal et al., 2022). It is of particular concern for people with underlying respiratory conditions, specifically those with cystic fibrosis or bronchiectasis (Bronson et al., 2021). The treatment of these infections is complicated because *M. abscessus* is intrinsically drug resistant, employing a variety of drug resistance mechanisms such as drug resistance genes, efflux pumps and an impermeable cell wall (Nessar et al., 2012). This results in lengthy ineffective treatment regimens of various antibiotics, often resulting in patients refusing the course of treatment (Haworth et al., 2017). Further to this, *M. abscessus* is comprised of 3 subspecies, these are *M. abscessus*, subsp. *abscessus*, *M. abscessus* subsp. *bolletii* and *M. abscessus* subsp. *massiliense* (Bryant et al., 2013). One of the main differences between the subspecies is due to the presence or absence of a functional *erm*(41) gene encoding inducible macrolide resistance. The *erm*(41) gene is present in *M. abscessus* subsp. *abscessus* and *bolletii* but is absent in *M. abscessus* subsp. *massiliense*, further limiting treatment options (Lee et al., 2015). Therefore, new and novel strategies to target these infections are urgently required. One area of renewed interest for microbial infections is manuka honey.

Manuka honey is a well-established antimicrobial, with a broad spectrum of activity that is attributed to the presence of methylglyoxal (Nolan et al., 2019). The established antimicrobial potential of manuka honey has resulted in the development of various medical grade honeys and has been further explored for its synergistic activity alongside antibiotics. The majority of these combinations have been explored for *Staphylococcus aureus* and *Pseudomonas aeruginosa*, demonstrating that antimicrobial activity of antibiotics can be greatly improved by the addition of manuka honey (Jenkins and Cooper, 2012a, Jenkins and Cooper, 2012b, Müller et al., 2013). This improved activity has also been demonstrated for *S. aureus* and *P. aeruginosa* biofilms (Campeau and Patel, 2014). The most interesting of these findings is the apparent reversal of antibiotic resistance by the addition of manuka honey, which has been observed for both *S. aureus* and *P. aeruginosa* (Rowena Jenkins and Cooper, 2012a; Rowena. Jenkins and Cooper, 2012b). It has been shown that manuka honey containing high levels of methylglyoxal can impact bacterial cell morphology, typically shortening the cell in *Bacillus subtilis, Escherichia coli* and *S. aureus* during lag phase but causing lengthening of the cell in *P. aeruginosa* (Lu et al., 2013). Condensing of the DNA was also observed for *B. subtilis*, *S. aureus* and *P. aeruginosa* after manuka honey exposure. A similar occurrence has also been observed in response to methylglyoxal alone, where methylglyoxal exposure resulted in shrinking and rounding of the cell as well as loss of fimbriae and flagella in *E. coli* and *B. subtilis* which ultimately caused loss of membrane integrity (Rabie et al., 2016). Although the mechanism of action behind manuka honeys antimicrobial activity has not yet been fully elucidated, the presence of methylglyoxal suggests that bacterial cell membrane disruption is likely to be one of the leading contributing factors. These findings show that manuka honey has great potential in the fight against antimicrobial resistant infections.

Recently, we published advances demonstrating that manuka honey is effective against *M. abscessus* and clinical isolates, as well as showing that manuka honey can improve the activity of amikacin (Nolan et al., 2022). Considering that some *M. abscessus* subspecies have macrolide resistance, there is potential to use manuka honey to regain susceptibility to them. One of the main antibiotics prescribed for the treatment of *M. abscessus* pulmonary infections is azithromycin, and although it is administered intravenously, it has approval to be used in a nebulised form, which further expands the potential of this combination, as therapeutic manuka honey use is considered to be limited to topical delivery. Another nebulised antibiotic used for treatment of cystic fibrosis infections is tobramycin, an aminoglycoside antibiotic with similar chemical structure to amikacin. It is therefore possible that by combining manuka honey and tobramycin improved antimicrobial activity against *M. abscessus* could be observed.

In this paper we demonstrate that the combination of manuka honey with azithromycin has the potential to lower the amount of antimicrobial required for improved activity against *M. abscessus* and the subspecies. We also show that tobramycin is not enhanced by the addition of manuka honey. Additionally, we demonstrate that by the addition of manuka honey to azithromycin, growth inhibition of the macrolide resistant *M. abscessus* subsp. *bolletii* can be achieved.

## 2 Materials and methods

### 2.1 Chemicals and reagents

All chemicals and reagents were obtained from Sigma-Aldrich or Melford Laboratories, unless otherwise stated. A total of 4 manuka honey samples were selected, each with a different MGO rating, MGO40 (Manuka Doctor, UK), MGO55 (ManukaPharm, UK), MGO70 (Manuka Doctor, UK) and MGO83 (Comvita, UK). All honey samples were stored in the dark at room temperature. Prior to testing, 1 g/mL stocks of honey in sterile distilled water were made and filtered in a 2 step filtration process using 0.8 µm filter to remove larger particulates and then sterilisation through a 0.22 µm filter.

### 2.2 Growth of *mycobacteria abscessus* cultures

The *M. abscessus* strains used for antimicrobial susceptibility testing were NCTC 13031 and 3 clinical isolates (159544, DC088A and DC088D) obtained from the Brighton and Sussex Medical School from patients with *M. abscessus* pulmonary infection, including cystic fibrosis and bronchiectasis patients. The 3 clinical isolates were the 3 subspecies, *M. abscessus* subsp. *abscessus* (159544), *M. abscessus* subsp. *bolletii* (DC088A) and *M. abscessus* subsp. *massiliense* (DC088D). *M. abscessus* cultures were grown from glycerol stocks (stored at −80 °C), in Middlebrook 7H9 broth, supplemented with glycerol (1 % w/v) and Tween80 (0.05 % w/v), at 37 °C, for 72 h in an orbital shaker at 180 rpm.

### 2.3 Checkerboard assay for manuka honey with azithromycin or tobramycin

A checkerboard assay was chosen to assess synergy between manuka honey samples and either azithromycin or tobramycin by modifying a broth microdilution assay and diluting one antimicrobial along the x axis and the other antimicrobial along the y axis. Both azithromycin and tobramycin were prepared in dimethyl sulfoxide (DMSO) to the final concentrations of 16, 8, 4, 2, 1, 0.5, 0.25, and 0 µg/mL along the x axis. Honey samples were prepared to final concentrations of 0.277, 0.237, 0.197, 0.157, 0.117, 0.077, 0.037 and 0 g/mL along the y axis. The plates were inoculated with OD_600nm_ = 0.1 adjusted *M. abscessus* cultures. These were then incubated at 37 °C for 96 h, with OD reads taken every 24 h. After 96 h, 5 µL of all wells were transferred onto Middlebrook 7H11 agar supplemented with glycerol (1 % w/v) and incubated at 37 °C for a further 72 h. The minimum inhibitory concentration (MIC) was determined as the minimum concentration required to inhibit the growth of *M. abscessus* and the minimum bactericidal concentration (MBC) was determined as the minimum concentration where no growth of *M. abscessus* was observed after plating out each condition on solid media.

## 3 Results

### 3.1 Azithromycin and manuka honey in combination against *M. abscessus* complex

The growth inhibition of *M. abscessus* and the subspecies were improved by the combination of manuka honey and azithromycin, resulting in lower concentrations of both antimicrobials, compared to each being used alone (Table 1). The improved activity was observed for all 4 manuka honeys tested, typically requiring 0.037 g/mL manuka honey when used alongside azithromycin, compared to 0.476 g/mL when used alone. Only the lowest graded manuka honey, MGO40, needed a higher concentration of 0.117 g/mL in combination with azithromycin for improved activity to be observed. However, this was still reduced compared to the concentration of manuka honey alone, which was 0.476 g/mL. Importantly, the addition of manuka honey reduced the concentration of azithromycin required for growth inhibition, regardless of subspecies. The most improved reduction of azithromycin concentration was observed for *M. abscessus* subsp. *bolletii*, which was reduced from >16 µg/mL to 4 µg/mL for MGO40 and MGO70.

**Table 1:** Minimum inhibitory concentrations of manuka honey and azithromycin both alone and in combination against *M. abscessus* and subspecies (n = 4).

*M. abscessus* isolate	Honey alone MIC (g/mL)	Honey combination MIC (g/mL)	Azithromycin alone MIC (µg/mL)	Azithromycin combination MIC (µg/mL)
MGO40				
NCTC 13031	0.476	0.037	4	1
subsp. *abscessus*	0.476	0.077	2	0.5
subsp. *bolletii*	0.476	0.117	>16	4
subsp. *massiliense*	0.476	0.037	1	0.5
MGO55				
NCTC 13031	0.476	0.037	4	1
subsp. *abscessus*	0.476	0.037	2	1
subsp. *bolletii*	0.476	0.037	>16	8
subsp. *massiliense*	0.476	0.037	1	0.5
MGO70				
NCTC 13031	0.476	0.037	4	0.5
subsp. *abscessus*	0.476	0.037	2	1
subsp. *bolletii*	0.476	0.037	>16	4
subsp. *massiliense*	0.476	0.037	2	0.5
MGO83				
NCTC 13031	0.476	0.037	2	0.5
subsp. *abscessus*	0.476	0.037	1	0.5
subsp. *bolletii*	0.476	0.037	>16	8
subsp. *massiliense*	0.476	0.037	1	0.25

**Fig. 1:**
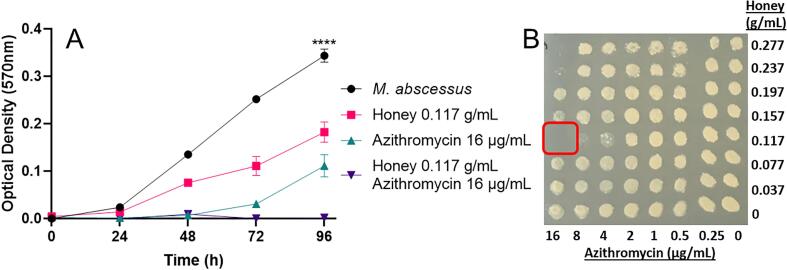
Improved inhibition and bactericidal activity of *M. abscessus* subsp. *bolletii* treated with manuka honey and azithromycin (n = 4). A) Growth curve of *M. abscessus* subsp. *bolletii* treated with 0.117 g/mL manuka honey and 16 µg/mL azithromycin. Growth inhibition can be seen for both manuka honey and azithromycin in combination. A one-way ANVOA identified a significant difference between all treatments, P=<0.0001. B) The growth of *M. abscessus* subsp. *bolletii* after transfer onto solid media. The red box indicates the absence of growth for the combination of 0.117 g/mL manuka honey and 16 µg/mL azithromycin only. Growth can be observed for all other concentrations (n = 4). (For interpretation of the references to colour in this figure legend, the reader is referred to the web version of this article.)

Improved bactericidal activity was also observed for the combination of azithromycin and manuka honey, compared to each antimicrobial used alone (Table 2). The two lower grade manuka honeys, MGO40 and MGO55, required 0.117 g/mL for improved bactericidal activity in combination with azithromycin, and a variation in concentration of azithromycin was required depending on the subspecies. The two higher grade manuka honeys, MGO70 and MGO83, showed improved bactericidal activity using only 0.037 g/mL when in combination with azithromycin. The concentration of azithromycin required for improved activity with MGO70 and MGO83 was reduced from >16 µg/mL to 4 µg/mL or less, depending on the subspecies. Interestingly, improved bactericidal activity was only observed for the combination of MGO40 and azithromycin against *M. abscessus* subsp. *bolletii*. The improved activity was only observed in response to 0.117 g/mL manuka honey and 16 µg/mL azithromycin (Fig. 1). There was no improved activity observed for the other manuka honeys tested against *M. abscessus* subsp. *bolletii*, which has been reported as no interaction (Table 2).

**Table 2:** Minimum bactericidal concentrations of manuka honey and azithromycin both alone and in combination against *M. abscessus* and subspecies (n = 4).

*M. abscessus* isolate	Honey alone MBC (g/mL)	Honey combination MBC (g/mL)	Azithromycin alone MBC (µg/mL)	Azithromycin combination MBC (µg/mL)
MGO40				
NCTC 13031	0.476	0.117	>16	16
subsp. *abscessus*	0.476	0.117	>16	4
subsp. *bolletii*	0.476	0.117	>16	16
subsp. *massiliense*	0.476	0.037	>16	0.5
MGO55				
NCTC 13031	0.476	0.117	>16	16
subsp. *abscessus*	0.476	0.117	>16	2
subsp. *bolletii*	0.476	No interaction	>16	No interaction
subsp. *massiliense*	0.476	0.037	>16	0.5
MGO70				
NCTC 13031	0.476	0.037	>16	4
subsp. *abscessus*	0.476	0.037	>16	1
subsp. *bolletii*	0.476	No interaction	>16	No interaction
subsp. *massiliense*	0.476	0.037	>16	1
MGO83				
NCTC 13031	0.476	0.037	>16	1
subsp. *abscessus*	0.476	0.037	>16	2
subsp. *bolletii*	0.476	No interaction	>16	No interaction
subsp. *massiliense*	0.476	0.037	>16	1

### 3.2 Tobramycin and manuka honey in combination against *M. abscessus* complex

Combinations of tobramycin with manuka honey did not show the same improvement in activity. No improved growth inhibition was observed for any combination of manuka honey with tobramycin tested against any of the *M. abscessus* strains tested. This was also the case for bactericidal activity, with no improved activity for any combination of manuka honey and tobramycin tested for any *M. abscessus* isolate tested.

## 4 Discussion

The rise of infections caused by *M. abscessus* complex is a growing problem, with particular concern for immunocompromised individuals (Abdelaal et al., 2022). Treatment options for pulmonary *M. abscessus* infections are limited, lengthy and often ineffective, urgently requiring novel strategies to target these infections. After establishing that manuka honey is effective against *M. abscessus* and clinical isolates, exploration into antibiotic-manuka honey combinations is an important next step for further informing potential treatments.

Improved antimicrobial activity against *M. abscessus* was observed in response to the macrolide antibiotic azithromycin with the addition of manuka honey. Typically, the concentration of manuka honey required for the improved activity of azithromycin was 0.037 g/mL manuka honey, apart from for MGO40 which required up to 0.117 g/mL depending on the subspecies tested. These concentrations are similar to the previous observations of the same manuka honeys used in combination with amikacin against the same *M. abscessus* strains (Nolan et al., 2022). With the addition of manuka honey the concentrations of azithromycin required for inhibition were reduced by twofold or more, such as 4 µg/mL to 1 µg/mL for *M. abscessus* NCTC. Bactericidal activity was also observed in response to azithromycin and manuka honey combinations for 3 of the *M. abscessus* isolates tested. Although all of the 4 manuka honeys exhibited bactericidal activity in combination with azithromycin, the 2 higher grades of manuka honey, MGO70 and MGO83, resulted in the most improved activity. This could be due to the higher MGO content, which has been shown to impact the bacterial cell membrane of *Escherichia coli*, causing shrinking and rounding of the cell (Rabie et al., 2016). This could allow for easier penetration into the cell membrane, resulting in improved antibiotic activity. Interestingly, bactericidal activity was observed for *M. abscessus* subsp. *bolletii* for one of the manuka honeys, MGO40, with azithromycin but only at the concentrations of 0.117 g/mL manuka honey and 16 µg/mL azithromycin (n = 4). Due to one of the main defining characteristics between the subspecies, inducible macrolide resistance, it was not surprising that bactericidal activity was not observed for the other manuka honeys in combination with azithromycin against *M. abscessus* subsp. *bolletii*. Conversely, improved bacteriostatic activity was observed for all of the manuka honeys tested against *M. abscessus* subsp. *bolletii*, suggesting there could be a potential interaction between azithromycin and manuka honey. Other than MGO concentration, it is unknown what specific differences were present between the manuka honeys. Therefore, it is hard to suggest why MGO40 in combination with azithromycin resulted in bactericidal activity compared to the other manuka honeys.

The combination of manuka honey and tobramycin did not exhibit improved activity at the concentrations tested. It was expected that activity observed previously for amikacin with manuka honey would also be observed with tobramycin since both are aminoglycoside antibiotics with similar chemical structures and mechanisms of action. However, no interactions were observed, bacteriostatic or bactericidal, for any concentration tested or any isolate in response to tobramycin with manuka honey. One of the key differences between amikacin and tobramycin is the presence of either a 2′-hydroxyl group or a 2′-amino group, respectively. It has previously been identified that aminoglycoside antibiotics with a 2′-amino group are less effective at inhibiting *M. abscessus* than those with the 2′-hydroxyl group. Interestingly, this was only observed for *M. abscessus* and both modifications were effective against *Mycobacterium smegmatis* (Maurer et al., 2014). This has been attributed to the presence of aminoglycoside acetyltransferases, more specifically aminoglycoside 2′-*N*-acetyltransferase (AAC(2′)), which have only been identified in Gram negative bacteria and mycobacterial species (Ramirez and Tolmasky, 2010). The AAC(2′) utilises acetyl-CoA to detoxify aminoglycoside antibiotics through acetylation of the 2′-amino group (Bacot-Davis et al., 2016). The lack of the amino group, replaced with a hydroxyl group in amikacin prevents the AAC(2′) from being effective. Furthermore, amikacin contains a (S)-4-amino-2-hydroxybutyrate (HABA) group at the N-1 position, which prevents hydrogen bonding with the enzyme and forces the enzyme to accommodate this group in a confirmation away from the active site (Bassenden et al., 2021). These differences in chemical structure could be the reason why tobramycin was not as effective as amikacin when combined with manuka honey. It is possible however, to improve the efficacy of tobramycin by the addition of other antibiotics, the most commonly suggested being β-lactams, however *M. abscessus* is intrinsically resistant to β-lactams so a β-lactamase would also be required (Sanz-García et al., 2019). Another possibility to improve its activity would be through the use of AAC inhibitors, which prevent the modification of the antibiotic, thus allowing it to remain effective (Jana and Deb, 2005, Labby and Garneau-Tsodikova, 2013).

Furthermore, it has previously, been identified that manuka honey and tobramycin work synergistically against *P. aeruginosa* isolates obtained from CF patients (Roberts et al., 2019). However, it was reported that subinhibitory concentrations resulted in antagonism in some cases, whereby the antimicrobial activity of the honey was ineffective and the additional sugar from the honey provided an alternative carbon source, thus allowing the bacterial isolates to overcome the antibiotic pressure. Suggesting that the low concentrations of manuka honey tested here were not sufficient enough to cause improved antimicrobial activity. Therefore, higher concentrations of manuka honey may be required for a synergistic interaction to be observed.

## Conclusion

Manuka honey is an effective antimicrobial against *M. abscessus* and has the potential to improve antibiotic activity. Combinations of azithromycin and manuka honey result in improved antimicrobial activity against *M. abscessus* isolates. The macrolide resistant *M. abscessus* subsp. *bolletii* exhibited growth inhibition in response to the combination and depending on the manuka honey bactericidal activity was also observed. Tobramycin did not have the same improved activity, with no changes in activity observed for any combination tested. This demonstrates that more antibiotic combinations should be tested with manuka honey to further the potential of manuka honey treatment.

## CRediT authorship contribution statement

**Victoria C. Nolan:** Conceptualization, Formal analysis, Methodology. **James Harrison:** Conceptualization, Formal analysis, Methodology. **Jonathan A.G. Cox:** Conceptualization, Formal analysis, Funding acquisition, Methodology, Supervision.

## Declaration of Competing Interest

The authors declare that they have no known competing financial interests or personal relationships that could have appeared to influence the work reported in this paper.
